# Enhancement of Mechanical and Barrier Property of Hemicellulose Film via Crosslinking with Sodium Trimetaphosphate

**DOI:** 10.3390/polym13060927

**Published:** 2021-03-17

**Authors:** Yuelong Zhao, Hui Sun, Biao Yang, Baomin Fan, Huijuan Zhang, Yunxuan Weng

**Affiliations:** 1 College of Chemistry and Materials Engineering, Beijing Technology and Business University, Beijing 100048, China; 13552134315@163.com (Y.Z.); ybiao@th.btbu.edu.cn (B.Y.); fanbaomin@btbu.edu.cn (B.F.); zhanghuijuan@btbu.edu.cn (H.Z.); 2 Beijing Key Laboratory of Quality Evaluation Technology for Hygiene and Safety of Plastics, Beijing Technology and Business University, Beijing 100048, China

**Keywords:** hemicellulose, sodium trimetaphosphate, crosslinking, barrier property

## Abstract

Hemicellulose is a kind of biopolymer with abundant resources and excellent biodegradability. Owing to its large number of polar hydroxyls, hemicellulose has a good barrier performance to nonpolar oxygen, making this biopolymer promising as food packaging material. Hydrophilic hydroxyls also make the polymer prone to water absorption, resulting in less satisfied strength especially under humid conditions. Thus, preparation of hemicellulose film with enhanced oxygen and water vapor barrier ability, as well as mechanical strength is still sought after. Herein, sodium trimetaphosphate (STMP) was used as esterification agent to form a crosslinked structure with hemicellulose through esterification reaction to render improved barrier performance by reducing the distance between molecular chains. The thus modified hemicellulose film achieved an oxygen permeability and water vapor permeability of 3.72 cm^3^ × μm × m^−2^ × d^−1^ × kPa^−1^ and 2.85 × 10^−10^ × g × m^−1^ × s^−1^ × Pa^−1^, respectively, at the lowest esterification agent addition of 10%. The crosslinked structure also brought good mechanical and thermal properties, with the tensile strength reaching 30 MPa, which is 118% higher than that of the hemicellulose film. Preliminary test of its application in apple preservation showed that the barrier film obtained can effectively slow down the oxidation and dehydration of apples, showing the prospect of application in the field of food packaging.

## 1. Introduction

Plastics as the youngest generation in material families are ubiquitous and have found tremendous application in all aspects of modern society. Most plastic products are petroleum-based and are nonrenewable by nature, thus giving rise to environmental issues when disposed of after service. Reports found that the world has accumulated more than 6 billion tons of plastic waste to date [1]. In light of the increasingly severe shortage of petrochemical resources and environmental pollution, the development of biodegradable bio-based materials has become the focus of current research. With respect to food packaging film, renewable lignocellulosic biomass has attracted much research attention due to the potential to replace petroleum-based film materials like polyethylene. Utilization of biomass as green packaging is expected to reduce the consumption of petrochemical resources and significantly reduce environmental pollution due to its biodegradability and renewability [2,3].

Bio-based food packaging film mainly derived from polysaccharide has been widely studied [4,5]. Among them, lignocellulose is rich in polysaccharide and has good biodegradability [6]. Hemicellulose is a type of lignocellulosic material, which is second only to cellulose in content. As a kind of heterogeneous polysaccharide composed of multiple glycogen structures [7], hemicellulose has excellent reproducibility, biodegradability and oxygen barrier [8]. However, due to the complexity and diversity of hemicellulose structure, the research on hemicellulose is still in its infancy [9,10,11]. The chemical modification methods of hemicellulose mainly include esterification [12,13,14], etherification [15,16,17], grafting [18,19] and crosslinking [20,21,22,23].

One of the primary requirements for a food packaging film is good barrier performance. To this end, various efforts have been made, including doping barrier particles such as montmorillonite [24], or by chemical reactions to make the interchains tighter [13,18,20,21] to improve film barrier so it can be comparable to traditional barrier materials. Among them, the films prepared by crosslinking reaction have good mechanical properties under the condition of excellent barrier performance. However, some of the chemicals used in previous work were less green, raising safety issues when used as packaging materials. In light of this, a nontoxic, edible chemical to produce film with lifted performance is sought after. In previous studies, citric acid crosslinked hemicelluloses were used to obtain film with excellent barrier properties [20]. However, the mechanical properties of the film obtained by this method are not improved much, and the amount of addition is larger when the film achieves good barrier properties. In this regard, to further improve the barrier performance of hemicellulose film, a safe and nontoxic sodium trimetaphosphate (STMP) commonly used in food additives was studied [25]. STMP has been utilized in the crosslinking reaction of a variety of polysaccharides [26,27].

This paper aimed to improve the barrier property of hemicellulose via formation of crosslinking structure using green crosslinker. In this attempt, STMP was chosen as a crosslinker to crosslink hemicellulose under alkaline conditions to form a tight network structure to reduce gas penetration. By using sorbitol as plasticizer [28] and polyvinyl alcohol (PVA) as reinforcing agent and cosubstrate [24], a series of barrier films with excellent properties was prepared by solution casting. The mechanical, thermal, barrier properties and wettabilities of the film were studied with regard to varying STMP contents. The thus modified hemicellulose film was applied in preservation test of apples.

## 2. Materials and Methods

### 2.1. Materials and Reagents

Poplar wood powder (particle size between 0.2 mm and 0.8 mm, from Hebei, China) was used. Its hemicellulose content was 30.5% and the main components of hemicellulose were *4-O*-methyl glucuronic acid xylose; the number-averaged molecular weight of hemicellulose was 7600 and the weight-averaged molecular weight was 24,000. Ethanol, sodium hydroxide and glacial acetic acid (Beijing Merida Technology Co., Ltd., Beijing, China), sodium trimetaphosphate (STMP), sodium chlorite (Shanghai Meryer Chemical Technology Co., Ltd., Shanghai, China), hydrochloric acid, sodium carbonate, toluene, sorbitol (AR, Sinopharm Chemical Reagent Co., Ltd., Shanghai, China) and PVA (1799, Shanghai Titan Technology Co., Ltd., Shanghai, China) were all used directly without further treatment.

### 2.2. Extraction of Poplar Hemicellulose

**Figure 1:**
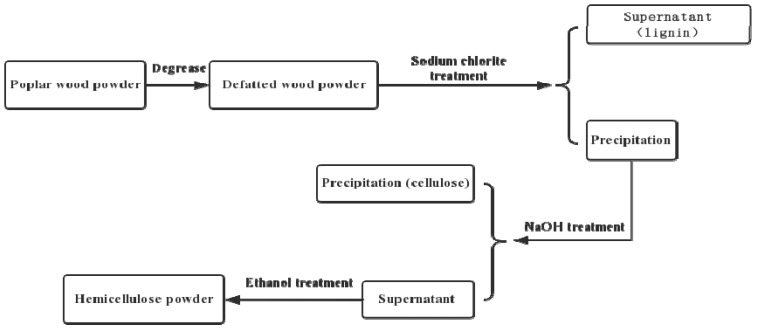
The flow chart of hemicellulose extraction.

Hemicellulose was extracted from the waste poplar powder by using the alkaline hydrolysis and alcohol precipitation [29]. Details of the procedure were described in a previous publication [20]. The overview of extraction process is shown in Figure 1.

### 2.3. Preparation of STMP Crosslinked Hemicellulose−Based Film

PVA was predissolved in water and the pH was adjusted to about 10 with Na_2_CO_3_ at 95 °C. After PVA was completely dissolved, hemicellulose and sorbitol were added and the mixture was stirred at 75 °C for 2 h. When a homogeneous solution was formed, STMP was added to allow reaction at 50 °C for 2 h.

After the reaction, the solution was sonicated for 10 min and defoamed in vacuum for 15 min. After this, the solution was poured into a polystyrene plastic culture dish (13 cm × 13 cm) for natural drying and film formation. The composition of each film is shown in Table 1.

### 2.4. Analytical Methods

**Infrared spectrum analysis (FT−IR):** The samples were analyzed on a Fourier infrared spectroscopy analyzer (iN10 MAX, Thermo Scientific Co., Ltd., Shanghai, China) using typical KBr tablet method. The scanning range of the spectrum was from 4000 cm^−1^ to 450 cm^−1^; the resolution was 4 cm^−1^ with 32 scans.

**Tensile test:** The film sample was cut into a rectangular specimen of 10 mm × 80 mm. A thickness gauge was used to measure the thickness of the film. The tensile test of the films was performed with a universal material testing machine (CMT6104, MTS Systems Co. Ltd., Wuhan, China). The initial distance was 60 mm and the stretching speed was 5.0 mm/min. Tensile strength and elongation at break values of the films were averaged over five specimens according to China national standard GB/T 1040.2-2006.

**Thermogravimetric analysis (TGA):** The thermal stability of a sample of about 5 mg was measured on a thermogravimetric analyzer (Q50, TA Instruments, New Castle, Pennsylvania, USA). At a rate of 20 °C/min, sample was heated from 40 to 700 °C. The thermal decomposition process was protected with a nitrogen atmosphere at a flow rate of 20 mL/min.

**Scanning electron microscopy (SEM) analysis:** The surface of the film sample was sprayed with gold, and the surface morphology of the films was observed on scanning electron microscope (Quanta FEG 250, FEI, Hillsboro, OR, USA). The acceleration voltage was 5 kV.

**Contact angle test:** Contact angles were measured using a contact angle tester (OCA35, DataPhysics Instruments GmbH, Beijing, China). First, 2 μL of water was dropped onto the surface of the sample. The droplet image was collected and the contact angle was calculated by using the software of the instrument. Five different locations on each sample were tested and the mean was taken as the static contact angle [30].

**Oxygen permeability (OP) measurement:** After the film samples were cut into wafers with a diameter of 10 cm, the oxygen permeability of the film was measured using VAC-V2 permeability analyzer (OX-TRAN 2/21, MOCON, Minneapolis, MN, USA). The test was carried out in accordance with the standard method as specified in Chinese National Standard GB/T 1038-2000. The measured temperature was 23 °C and the relative humidity (RH) was 50%. The average value of each result was taken on the three samples.

**Water vapor permeability (WVP) measurement:** The water vapor permeability was measured by the weighing method commonly used in literature and calculated using Equation (1) [13,31]. The films were sealed in a container containing dry silica gel. Next, the containers were placed in a desiccator containing water and weighed regularly every 24 h for 7 d. Each result was averaged over three specimens.

(1) $$\mathrm{WVP}=\frac{w\times L}{t\times A\times \Delta p}$$

where *w* is the weight gained (g), *L* is the film thickness (m), *t* is the elapsed time (s), *A* is the film permeation area (m^2^), Δ*p* is 2339 Pa at 20 °C.

**Test of film in apple preservation:** Apple pieces of roughly the same size (approximately 1/16th of an apple) were kept in three 50 mL beakers, two of which were sealed with hemicellulose film (HC) and sodium trimetaphosphate crosslinked hemicellulose film (STMP−10), respectively. As control, the third beaker was not covered with any film. The changes in the appearance of apples were visually observed.

## 3. Results and Discussion

**Figure 2:**
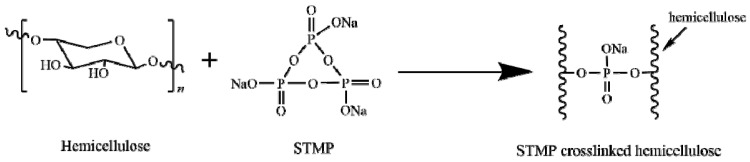
Crosslinking reaction of hemicellulose with sodium trimetaphosphate (STMP).

STMP hydrolyzes under alkaline conditions and can be esterified with the hydroxyl groups on the hemicellulose. Therefore, the hemicellulose film can form a network crosslinking structure by using STMP as the crosslinking agent, as shown in Figure 2.

### 3.1. Structure Analysis of STMP Crosslinked Hemicellulose Films

**Figure 3:**
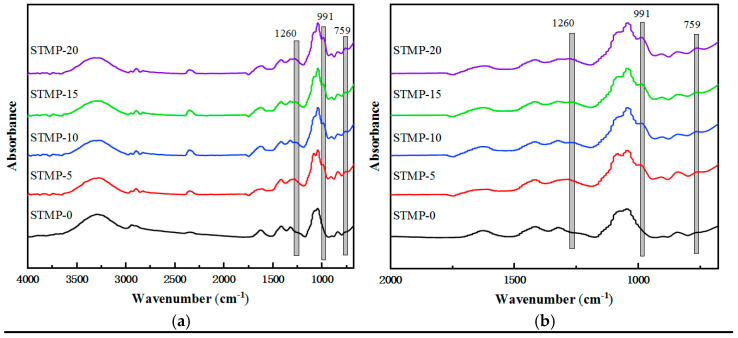
(**a**)FT−IR spectra of the STMP crosslinked hemicellulose films; (**b**) Fingerprint region of the FT−IR spectrum of STMP crosslinked hemicellulose films

The FT−IR spectra of the STMP crosslinked hemicellulose films are displayed in Figure 3. As shown in the figure, each film with varying STMP addition has obvious stretching vibration peak of fatty alcohol (–OH) at about 3300 cm^−1^, and the stretching vibration peak of alkane C–H at about 2900 cm^−1^. At 1050 cm^−1^, the stretching vibration peaks of C–O bond and C–C bond in hemicellulose or the bending vibration peaks of C–O (H) on sugar ring were observed, which was consistent with the structural characteristics of hemicellulose film reported in literature [15,20,32]. After the addition of STMP, three characteristic peaks of P/O bond appeared at 1260 cm^−1^, 991 cm^−1^ and 759 cm^−1^, and became more obvious with the increase of STMP addition, indicating the successful crosslinking of hemicellulose by STMP.

### 3.2. Mechanical Properties of STMP Crosslinked Hemicellulose Film

**Figure 4:**
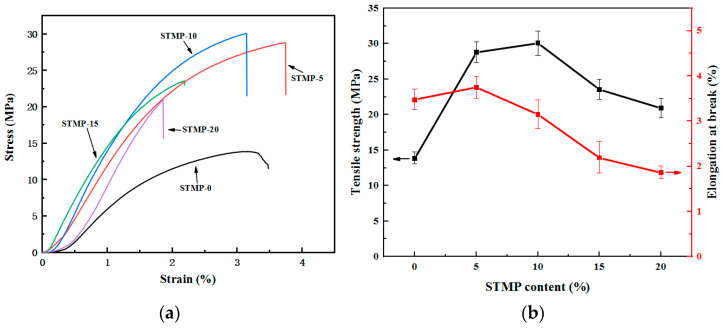
Mechanical properties of STMP crosslinked hemicellulose films (**a**) stress–strain curves; (**b**) mechanical properties (Black axis: Tensile strength, red axis: Elongation at break) at different STMP contents.

The test results of STMP crosslinked hemicellulose films are shown in Table 2 and Figure 4. It is seen that no obvious yield phenomenon of hemicellulose film was observed during tensile process. The tensile strength of the hemicellulose film was found to increase with increasing the STMP addition in the initial stage. The tensile strength of the hemicellulose film with 10% STMP was up to 30.08 MPa, which was 118% higher than that of the unmodified hemicellulose film. The rigidity of the film was gradually enhanced and the maximum elastic modulus reached 1512 MPa. This is mainly because STMP crosslinked hemicellulose to form a network structure, resulting in enhanced intermolecular force, and thus significantly improved tensile strength [33,34]. At higher STMP loadings, i.e., greater than 15%, the tensile strength and the elastic modulus film gradually decreased. This observation is mainly due to the gelation phenomenon in some places caused by the excessive addition of STMP, which leads to the decline of mechanical properties [35]. The elongation at break for films with 20% STMP addition was determined to be 1.86%, much lower than 3.48% for unmodified film. This variation in elongation at break in turn was consistent with the fact that the hemicellulose was crosslinked. It can be inferred that the hemicellulose film can be crosslinked completely at STMP addition level of about 10%, while the hemicellulose film will be over-crosslinked after 15% STMP loading. In addition, Sreedhar et al. [36] suggested that the strength of the crosslinked film was not only dependent on the degree of crosslinking, but also on the amount of electrostatic interaction. The decrease of film strength may be due to the fact that when the concentration of crosslinking agent is too high, there are more negative charges on the polymer matrix, resulting in electrostatic repulsion, leading to larger distance between molecules and decreased binding force, thus reduced strength.

### 3.3. Thermal Stability Analysis of STMP Crosslinked Hemicellulose Films

**Figure 5:**
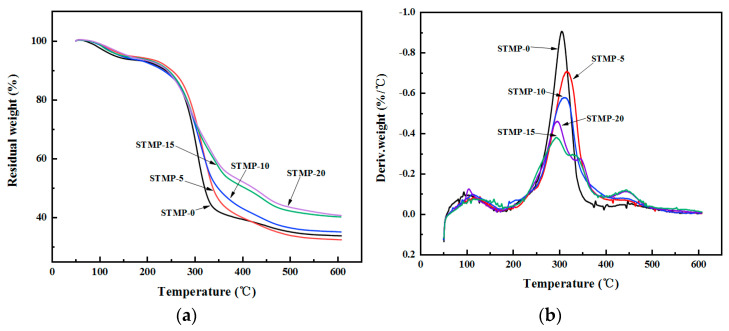
The thermal stabilities of STMP crosslinked hemicellulose films (**a**) thermogravimetric analysis (TGA) curves, (**b**) derivative thermogravimetry (DTG) curves.

The thermal stability of STMP crosslinked hemicellulose films is shown in Table 3 and Figure 5. It is seen from Figure 5a that the films displayed mainly three weight loss stages, i.e., 60~160 °C, 160~370 °C and 370~500 °C. The weight loss at 60~160 °C was mainly caused by the evaporation of residual water in the material and the adsorption of a small amount of water vapor [37]. With the increase of STMP contents, the weight loss at 60~160 °C decreased gradually, indicating that the water absorption of the material decreased, which is in accordance with the fact that the hemicellulose crosslinking reaction occurred. The main weight loss stage of the film was observed at 160~370 °C which is due to the rupture of C–O bond and C=O bond on the side chain of hemicellulose; while the weight loss stage of 370~550 °C originated from the rupture of C–C main chain on the hemicellulose skeleton, namely the carbonization process [20]. The carbon residue rate of the film increased with the increase of crosslinking degree, up to 40% at 600 °C.

As shown in Table 3 and Figure 5b, with the increase of STMP content, the temperature corresponding to the peak thermal decomposition rate of the crosslinked hemicellulose film in the second stage increased from 304.41 to 345.45 °C, and the thermal decomposition rate decreased, indicating that the thermal performance of the crosslinked hemicellulose film was improved. The increase of thermal decomposition temperature and the decrease of maximum thermal mass loss rate were mainly due to the tight structure induced by crosslinking [38]. When the addition amount of STMP was higher than 15%, two thermal decomposition rate peaks appeared (*T*_max2_ and *T*_max3_). This was mainly due to the decomposition of the excessive STMP that occurred first, and subsequent breaking of the side chain of the polymer, which is consistent with the analysis of the tensile test results.

### 3.4. Surface Morphology Analysis of STMP Crosslinked Hemicellulose Film

**Figure 6:**
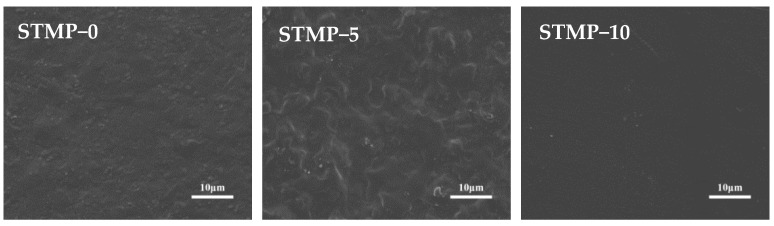

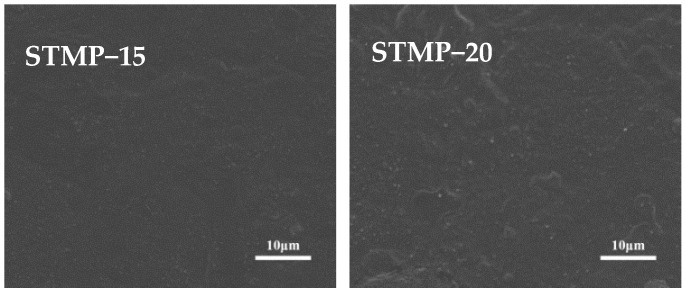
Scanning electron microscopy (SEM) images of STMP crosslinked hemicellulose films.

The surface morphology and structure of STMP crosslinked hemicellulose films were observed by SEM, as shown in Figure 6. The surface roughness of hemicellulose film decreased first and then increased after adding STMP. When STMP content was 10%, the surface of the film was the smoothest and tightest, with no gaps, cracks or other defects, and the molecular chains were closely stacking, indicating that the bonding between each component was stable through hydrogen bonds and van der Waals forces. It could be concluded that the crosslinking reaction at this time was more complete. However, when the amount of crosslinking agent is more than 15%, some granular substances will appear on the film surface, which is mainly because the excessive addition of crosslinking agent at this time leads to the increase of the roughness of the film surface.

The main reason for this phenomenon could be that the amount of STMP is not enough to crosslink hemicellulose completely, so that the molecular chain is relatively loose, and the excessive STMP cannot continue to participate in the crosslinking reaction and disperse uniformly in the film matrix, resulting in the increasing surface roughness of the film, and even the appearance of some micropores because of the gel phenomenon [35].

### 3.5. Surface Wettability Analysis of STMP Crosslinked Hemicellulose Film

**Figure 7:**
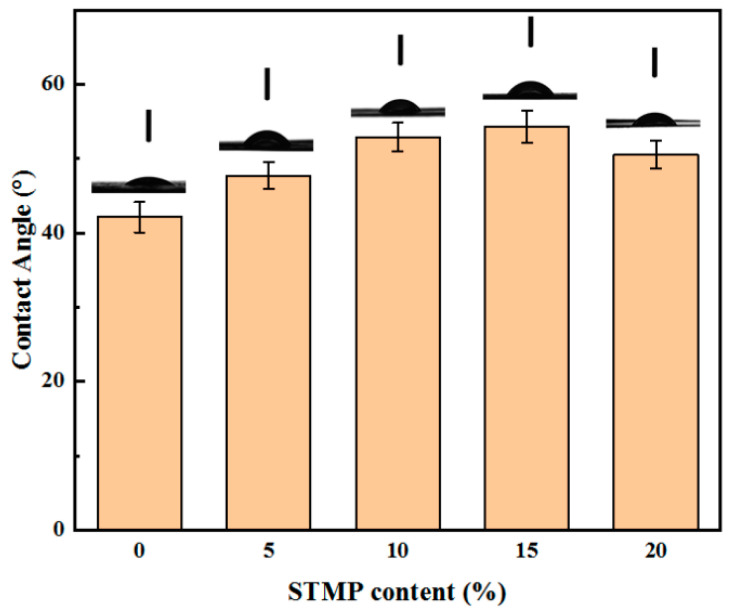
Contact angle results of STMP crosslinked hemicellulose films.

For assessment of surface wettability, pure water was used as the detection liquid. The contact angles were measured to be 42.1°, 47.7°, 52.9°, 54.3° and 50.5° for STMP−0, STMP−5, STMP−10, STMP−15 and STMP−20, respectively, as shown in Figure 7.

As can be seen from Figure 7, the contact angle of the film increased first and then decreased with rising STMP content. When the addition amount was 15%, the contact angle reached 54.3°. Similarly, this was mainly because STMP and the hydroxyl group of hemicellulose underwent esterification reaction, resulting in decreased number of hydroxyls, decreased polarity and decreased surface energy. Due to the repulsive force, the nonpolar surface produced a larger contact angle, which caused the reduced hydrophilicity [39]. The decrease in hydrophilicity contributed to reducing mechanical properties loss under wet environment and reducing the water vapor permeation of the film [40].

### 3.6. Oxygen Barrier Properties of STMP Crosslinked Hemicellulose Film

The oxygen permeability of hemicellulose films with different STMP content at 23 °C and 50% RH is shown in Table 4.

The main functions of packaging materials include the prevention of oxidation and deterioration caused by oxygen penetration, so packaging materials need to have low oxygen permeability. As shown in Table 4, compared with the unmodified hemicellulose film, the oxygen permeability of the hemicellulose film with STMP was first decreased and then slightly increased, with the lowest value of 3.72 cm^3^ × μm × m^−2^ × d^−1^ × kPa^−1^ observed at 10% STMP content, a reduction by 64% compared with the unmodified film. At the same crosslinker level (20%), oxygen barrier performance was further improved compared to hemicellulose film crosslinked with citric acid (with oxygen permeability of 5.4 cm^3^ × μm × m^−2^ × d^−1^ × kPa^−1^). This oxygen permeability reached the level of traditional petroleum-based barrier film [41], e.g., 2.88 cm^3^ × μm × m^−2^ × d^−1^ × kPa^−1^ for EVOH, and is much lower than that of biodegradable polymer polylactic acid [42], i.e., 160 cm^3^ × μm × m^−2^ × d^−1^ × kPa^−1^. The main reason is that the crosslinking of STMP and hemicellulose leads to the formation of crosslinking network structure, resulting in less intermolecular space, more compact film and thus lower oxygen permeability [34]. At the same time, there is less free space inside the film, making it harder for oxygen to enter the film [43]. When the STMP addition amount is more than 15%, over-crosslinking may cause some holes to appear on the film surface, thus slightly increasing the oxygen permeability [35]. Meanwhile, the electrostatic effect caused by the high concentration of crosslinking agent will enlarge the distance of molecular chain and improve the permeability of oxygen [36], which is consistent with the inference in the tensile property test, thermal property test and surface morphology test above.

### 3.7. Water Vapor Barrier Properties of STMP Crosslinked Hemicellulose Film

In the case of food packaging, lower water vapor permeability prevents water loss from the food, rendering better preservation. The test results of water vapor permeability of hemicellulose films with different STMP contents at 20 °C under about 100% RH are summarized in Table 5. As shown in the table, the variation trend of water vapor permeability is the same as that of oxygen permeability discussed above. With the addition of STMP, the water vapor permeability of the hemicellulose film can reach 2.85 × 10^−10^ g × m^−1^ × s^−1^ × Pa^−1^ (10% STMP content), which is reduced by 41% compared with that of the unmodified hemicellulose film. The main reason for the decrease of water vapor permeability of hemicellulose film is the same as that of oxygen permeability, i.e., a crosslinking reaction occurred to produce a denser film, leading to decreased oxygen and water vapor permeability. In the case of higher STMP loading, excessive crosslinking agent leads to gel formation, resulting in holes, and thus slightly increased water vapor permeability. This explanation agrees well with the analysis of the SEM results.

### 3.8. Test of STMP Crosslinked Hemicellulose Film for Apple Preservation

**Figure 8:**
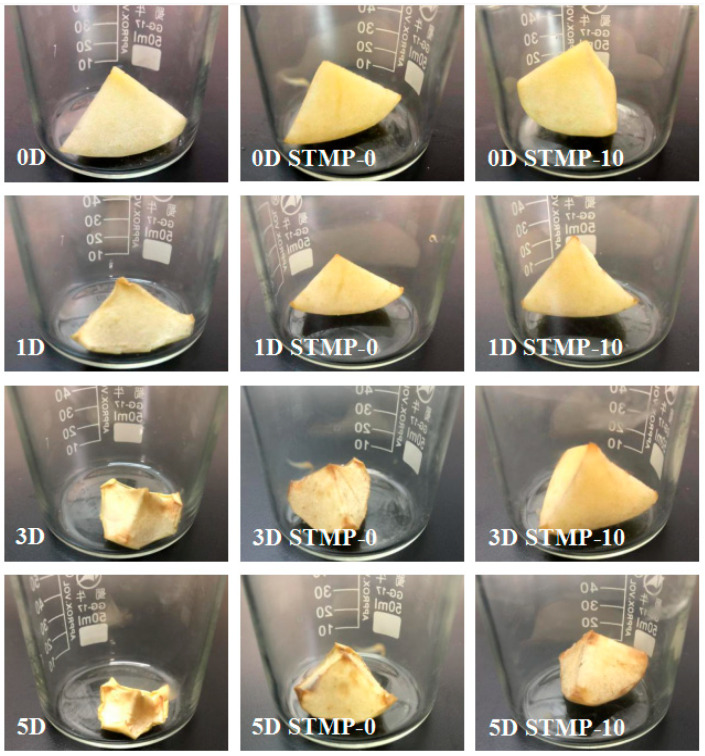
Appearance (oxidation and dehydration status) evolution of apple sample exposed to air (left column), stored in beaker sealed with hemicellulose film (middle column) and STMP crosslinked hemicellulose film (right column) with time (rows 1–4 corresponding to 0 d, 1d, 3 d and 5d, respectively), showing the potential of modified hemicellulose film in fruit packaging.

Apple is prone to oxidize and dehydrate in air. The barrier performance of STMP crosslinked hemicellulose film was evaluated by comparing the daily changes of apples in the air, in hemicellulose film and in STMP crosslinked hemicellulose film via visual observation. As shown in Figure 8, in the beginning of the test, all apple samples were full of moisture and uniform in color. After 1 d, the apples in the air were slightly discolored, which was caused by oxygen oxidation in the air, and began to lose water as indicated by the withered quality, while apples in the hemicellulose film and STMP crosslinked hemicellulose film were still full of water and had no signs of discoloration. After 3 d, the apples in the air suffered severe water loss and oxidation discoloration. The apples in the hemicellulose film also began to undergo oxidation discoloration and showed slight water loss, while the apples in the STMP crosslinked hemicellulose film were still well-hydrated with no sign of discoloration. Finally, on the fifth day, apples in the air dried, shriveled and discolored, indicating loss of most water and oxidization. The apples in the hemicellulose film were further oxidized, and the apples in the STMP crosslinked film also began to show oxidation discoloration and water loss. The above observation showed that the STMP crosslinked film and hemicellulose film can significantly cut off the oxygen and water vapor, and the barrier performance of the hemicellulose film crosslinked by STMP was significantly enhanced compared with that of the unmodified hemicellulose film. The apple which was dehydrated and oxidized in the air on day 1 can be preserved until day 3 with full moisture and uniform color if otherwise packed in modified hemicellulose film, indicating the potential to protect food from oxidation and dehydration.

## 4. Conclusions

Hemicellulose film with improved mechanical, thermal and barrier properties was prepared by using STMP as crosslinking agent for hemicellulose via esterification. At an STMP mass fraction of 10%, the film showed the best comprehensive performance. The formation of crosslinking structure makes the tensile strength reach up to 30.08 MPa, 118% higher than that of the unmodified hemicellulose film; the oxygen permeability and water vapor permeability could reach 3.72 cm^3^ × μm × m^−2^ × d^−1^ × kPa^−1^ and 2.85 × 10^−10^ g × m^−1^ × s^−1^ × Pa^−1^, respectively, due to the formation of a dense structure. The barrier performance of the modified hemicellulose film was evaluated and verified via a test of preservation of apple where changes, i.e., water loss, discoloration with time were visually observed. The preliminary results indicated a good prospect for sodium trimetaphosphate crosslinked hemicellulose film in the field of food packaging.

## Figures and Tables

**Table 1:** Nomenclature and composition of films.

Sample	STMP Mass Fraction (%)	Mass of Hemicellulose (g)	Mass of PVA (g)	Mass of Sorbitol (g)
STMP−0	0	0.90	0.30	0.30
STMP−5	5	0.90	0.30	0.30
STMP−10	10	0.90	0.30	0.30
STMP−15	15	0.90	0.30	0.30
STMP−20	20	0.90	0.30	0.30

**Table 2:** Tensile test results of the STMP crosslinked hemicellulose films.

Sample	Thickness (μm)	Tensile Strength (MPa)	Elongation at Break (%)	Modulus of Elasticity (MPa)
STMP−0	52 ± 3	13.85 ± 0.84	3.48 ± 0.23	827 ± 85
STMP−5	56 ± 2	28.79 ± 1.43	3.75 ± 0.24	929 ± 93
STMP−10	61 ± 2	30.08 ± 1.72	3.15 ± 0.32	1512 ± 116
STMP−15	62 ± 3	23.56 ± 1.38	2.19 ± 0.35	1473 ± 133
STMP−20	64 ± 1	20.92 ± 1.35	1.86 ± 0.14	958 ± 79

**Table 3:** Thermal properties of STMP crosslinked hemicellulose films.

Sample	*T*_max_ (°C)				Carbon Residue Rate at 600 °C (%)
	*T*_max1_ (°C)	*T*_max2_ (°C)	*T*_max3_ (°C)	*T*_max4_ (°C)	
STMP−0	103.59	−	304.41	446.96	33.84
STMP−5	114.41	−	316.25	441.48	32.51
STMP−10	113.37	−	311.96	442.52	35.17
STMP−15	115.45	293.58	334.63	443.70	40.27
STMP−20	107.89	294.78	345.45	442.85	40.78

**Table 4:** Oxygen permeability of STMP crosslinked hemicellulose films^1.^

Sample	Oxygen Permeability ^1^ (cm^3^ × μm × m^−2^ × d^−1^ × kPa^−1^)
STMP−0	10.46 ±0.38
STMP−5	6.71 ± 0.29
STMP−10	3.72 ± 0.11
STMP−15	3.76 ± 0.20
STMP−20	3.98 ± 0.08

^1^ Test conditions: 23 °C, 50% RH.

**Table 5:** Water vapor permeability of STMP crosslinked hemicellulose films^1.^

Sample	Water Vapor Permeability ^1^ (10^−10^ g × m^−1^ × s^−1^ × Pa^−1^)
STMP−0	4.82 ± 0.63
STMP−5	3.51 ± 0.25
STMP−10	2.85 ± 0.50
STMP−15	2.94 ± 0.47
STMP−20	3.19 ± 0.53

^1^ Test conditions: 20 °C, 100% RH.
